# Recent Developments in Psychopharmaceutical Approaches to Treating Female Sexual Interest and Arousal Disorder

**DOI:** 10.1007/s11930-017-0124-3

**Published:** 2017-10-19

**Authors:** Stephanie Both

**Affiliations:** Department of Psychosomatic Gynecology and Sexology, Leiden University Medical Center, Albinusdreef 2, PO Box 9600, 2300 RC Leiden, The Netherlands

**Keywords:** Sexual desire, Sexual arousal, Sexual dysfunctions, Pharmacological

## Abstract

**Purpose of Review:**

This review summarizes the recent literature and empirical studies on psychopharmacological approaches to treating female sexual interest/arousal disorder (FSIAD).

**Recent Findings:**

Several new drugs for FSIAD that are intended to increase sexual responsiveness by influencing central excitatory and inhibitory neuromodulatory processes are under development. Studies on flibanserin resulted in the first approved medication for the treatment of low sexual desire in premenopausal women. New drugs under development are testosterone combined with sildenafil or buspiron, bremelanotide, BP101, and nasal testosterone (TBS-2).

**Summary:**

Although pharmacological enhancement of sexual responsiveness may be potentially helpful in the treatment of FSIAD, the observed effects of flibanserin and other new drugs under development seem limited in terms of clinical significance. Given the multifactorial character of FSIAD, it may be important to integrate psychopharmacological treatment with sex therapy for optimal treatment efficacy.

## Definition and Prevalence of Female Sexual Interest and Arousal Disorder

Female sexual interest/arousal disorder (FSIAD) is characterized by a lack of, or significantly reduced, sexual interest and/or arousal [1]. It is manifested by at least three of the following symptoms: (1) absent or reduced interest in sexual activity, (2) absent or reduced erotic thoughts or fantasies, (3) no or reduced initiation of sexual activity and typical unreceptivity to a partner’s attempts to initiate, (4) absent or reduced sexual excitement or pleasure during sexual activity, (5) absent or reduced sexual interest or arousal in response to any internal or external erotic cue, and (6) absent or reduced genital or nongenital sensations during sexual activity. For a diagnosis to be given, the symptoms must be present for a minimum duration of approximately 6 months, and they must cause clinically significant distress in the individual. Furthermore, the complaints should not be better explained by a nonsexual mental disorder, severe relationship distress, or other significant stressors. Also, they must not be exclusively associated with the consequences of a medical condition, and when substance or medication use can explain the complaints, the diagnosis substance or medication-induced sexual dysfunction should be made.

In clinical practice, the complaint of a lack of sexual interest or arousal is seldom presented by single women. Usually, the complaint comes from women in a steady relationship and is related to differences in sexual desire between her and her partner. In the DSM-5, it is explicitly stated that a desire discrepancy, in which a woman has lower desire for sexual activity than her partner, is not sufficient to diagnose FSIAD. Also, the DSM notes that a normative decline in sexual thoughts and response with age should be taken into account. However, it is unclear how much disinterest or reduction in interest a woman needs to show in order to qualify for a sexual interest or arousal disorder. The DSM lacks objective criteria, which means that the diagnosis has to be based on the subjective judgment of the clinician, who also must consider factors that might influence sexual functioning, such as gender or age. Gender is known to be an important factor. On the basis of a review of a large number of studies on differences in sexual motivation between men and women, Baumeister, Catanese, and Vohs [2] concluded that women generally have lower and less frequent sexual motivation than men. Women masturbate less, fantasize less about sex, and have less frequent desire for sex than men.

Little or no sexual desire is the most common sexual problem reported by women. In prevalence studies, the occurrence of symptoms of sexual dysfunction and also the degree of distress caused by the problems were investigated [3–5]. In these studies, following the DSM-IV-R, low sexual desire and sexual arousal were considered as separate disorders. Although the studies differed in their diagnostic criteria and study methods, the prevalence of low sexual desire in the general population of women was consistently found to be about 20 to 30%. When distress about the low desire was used as a necessary criterion for dysfunction, the prevalence rates decreased by an average of one half. The prevalence of sexual arousal problems across studies was between 11 and 31%, but in combination with the criterion of distress, these rates also decreased sharply. These findings show that sexual interest and arousal problems are fairly common in women, but that only a proportion of the women experience distress from them and thus would qualify for a sexual dysfunction diagnosis.

## Underlying Mechanisms of Sexual Interest and Arousal

What do we know about the underlying mechanisms of sexual interest and arousal? According to modern incentive motivation theories, sexual interest and arousal are the result of an interplay between a person’s internal sexual response system and external stimuli (incentives) that activate this system [6]. The sensitivity of the sexual response system plays a role in conjunction with the meaning and intensity of the real or imaginary stimuli. Sexual motivation is not seen as a biological drive that comes from within—a drive that can be strong or weak —but as a state that manifests when certain conditions are met. The conditions necessary to activate the sexual process have three parts: (1) there must be an intact system that enables sexual responsiveness, (2) stimuli with a sexual meaning must be present that can activate the sexual system, and (3) the circumstances must be suitable to pursue sexual activity [7]. In this process, motivation starts to emerge and becomes stronger as the three conditions are met to a greater and greater degree. In contrast, if one or more of these conditions are lacking or absent, then the sexual motivation process breaks down. According to the incentive motivation model, biological as well as psychological factors can facilitate or hinder the activation of the sexual response system. Hormonal disorders can, for example, decrease the sensitivity of the sexual system, otherwise referred to as “arousability” or “sexual responsiveness” [8], and cognitive processes, such as negative thoughts or distraction, can suppress genital and subjective sexual arousal [9].

It is important to note that in the initiation of sexual activity, several motives can play a role. In a large group of study participants, Meston and Buss created an inventory of people’s motives to have sex with a partner [10]. Men and women reported a wide variety of motives, such as experiencing physical pleasure, showing affection, satisfying their partner, relieving boredom, or fulfilling a perceived obligation. Although the top 10 motives of the men and women were closely matched, the men were more inclined towards physical motives, such as seeing an attractive body, whereas the women were more inclined towards relational motives, such as showing love. In the female sexual response model developed by Basson, the need for intimacy plays an important role as a motive for sexual activity [11]. Basson emphasizes that, particularly in long-term relationships, a woman’s willingness to be sexual derives from her wish for intimacy and that this sexual receptivity can lead to sexual arousal and sexual desire. The rewarding value of the sexual interaction, in terms of sexual satisfaction and intimacy, then determines the extent to which the woman will be receptive to sexual stimuli in the future.

Studies in animal models of sexual function have revealed the brain and central nervous system pathways involved in sexual responding [12]. Based on these studies, sexual responsiveness is thought to be influenced by the interplay between excitatory and inhibitory neuromodulatory processes. In excitatory processes, testosterone, dopamine, norepinephrine, oxytocin, and melanocortins are thought to be important, while inhibitory processes seem to be regulated by serotonin, opioids, and endocannabinoids. Sexual interest is specifically regulated by the brain’s reward system circuitry, with a central role of the nucleus accumbens and its dopaminergic input pathway. The reward system circuitry is involved in motivational responses to various rewards such as food, drugs, and sex. The system is interconnected with the hypothalamus that integrates incentive signals and prepares the body for action. In this circuit, the prefrontal cortex is involved in the signaling of reward and in the regulation of responses. An imbalance in excitatory and inhibitory processes (e.g., hyperactive inhibitory or hypoactive excitatory responses) or a combination of both may underly sexual dysfunction such as low sexual interest and arousal [13••].

To instigate appetitive motivational responses, a sensory stimulus has to be assessed as rewarding. This evaluation is done within milliseconds, before one is consciously aware of it [14, 15]. Although there are stimuli that, by nature, result in sexual responses and pleasurable feelings in most people—such as stroking the genitals—many sexual stimuli will derive their meaning from associative learning processes such as classical and operant conditioning [16, 17]. We learn by experience what is rewarding. When an encounter with a stimulus has positive consequences and results in pleasure, this stimulus will be tagged as rewarding [18]. Learning about sexual stimuli generally leads to positive associations, but stimuli can also become associated with negative emotions, such as in sexual abuse [19]. A particular perfume for example, can be sexually attractive for one person because it is associated with a very pleasurable sexual experience, while it can elicit strong aversion in another person, because it is associated with a sexual abuse experience. The potential of stimuli that are able to evoke sexual interest and arousal will depend on the sexual learning history of the individual. If a woman has mainly negative experiences with sex, there may be no or only few stimuli that can instigate the expectation of sexual reward. Taken together, multiple factors play a role in sexual interest and arousal, and apart from the sensitivity of the brain and central nervous system pathways involved in sexual responding, cognitive and relational factors are important to consider in FSIAD [20].

## Recent Developments in Psychopharmacological Treatment of FSIAD

Obviously, influencing the excitatory and inhibitory neuromodulatory processes affecting sexual responsiveness by psychopharmacological drugs may be a tool to treat FSIAD. The search for drugs that can facilitate sexual interest and arousal—also called aphrodisiacs—has a long history. In this section, recent research, published since 2010, directed on the development of drugs to facilitate female sexual interest and arousal will be reviewed. The focus will be on studies on flibanserin, testosterone combined with sildenafil or buspiron, bremelanotide, BP101 (a synthetic peptide molecule), and nasal testosterone (TBS-2). In addition, recent research on the effect of d-cycloserine, a memory-enhancing drug, on sexual conditioning will be discussed.

### Flibanserin

After previous denial, in 2015, the American Food and Drug Administration (FDA) approved flibanserin (brand name Addyi) for the treatment of low sexual desire in premenopausal women. Flibanserin has mixed effects on the serotonergic and dopaminergic neurotransmitter systems and was initially developed as an anti-depressant, and later tested for pro-sexual effects. In a number of large trials in women with a diagnosis of hypoactive sexual desire disorder, it was shown that the use of flibanserin (compared to placebo) resulted in a significantly larger increase in the number of monthly so-called “satisfying sexual events” (SSEs) [21–23]. Although statistically significant, the effects of flibanserin were small; across the phase 3 studies, an increase relative to placebo of 1 to 1.5 SSEs a month was observed [24••]. Also, it is a drug that has to be used daily, with side effects such as dizziness, somnolence, nausea, and fatigue and it should not be used in combination with alcohol. The effects of long-term use are yet unknown. Based on the limited pro-sexual effect, the side effects, and the lack of data on safety with long-term use, there has been a vehement discussion about the approval of the drug [25]. Interestingly, a currently running study by Goldstein et al., registered on clinicaltrials.gov (https://clinicaltrials.gov/ct2/show/NCT02714049), intends to investigate whether flibanserin in combination with sex therapy may be more effective than flibanserin alone. The study is stated to include women with “biologic-based” hypoactive sexual desire disorder, but specific criteria for this diagnosis are not given. The study design involves an 8-week run-in period of flibanserin to differentiate responders from nonresponders. After that, the responders are randomized to receive 12 weeks flibanserin plus sex therapy or flibanserin only. The sex therapy condition involves nine sessions, in person or on the phone; unfortunately, the content is not described.

### Testosterone plus Sildenafil (Lybrido) and Testosterone plus Buspiron (Lybridos)

Inspired by the dual control model of sexual response [26], Tuiten and colleagues reasoned that low sexual desire in women may be due to either a relative insensitive brain system for sexual cues, or to enhanced activity of sexual inhibitory mechanisms [27•]. This distinction was taken into account in the design of pharmacotherapies for low sexual desire in women. A combination of testosterone (T, 0.5 mg sublingual) plus a phosphodiesterase type 5 inhibitor (sildenafil, 50 mg) was developed for women with low sensitivity to sexual cues [28]. In addition, a combination of T with a serotonin_1A_ receptor agonist (buspiron, 10 mg), intended to decrease sexual inhibition, was developed for women more inclined to sexual inhibition [29]. Both drugs are meant to be used on demand, 4 h before the wanted effects—an advantage because it addresses the potential safety concerns of prolonged use of androgens in women. In the first test of the drugs, 56 pre- and postmenopausal women with hypoactive sexual desire disorder (HSDD, based on DSM-IV-TR) participated in a complex, randomized, doubleblind, placebo-controlled cross-over design [30]. All women underwent three medication regimes: placebo, T + sildenafil, and T + buspiron, each for 4 weeks and completed an emotional Stroop task to measure preconscious attentional bias for sexual cues. During this task, sexual or neutral words were presented in color, very briefly (only 26 ms), and were immediately followed by randomly cut and reassembled letters in the same color (the masking stimuli). Women had to name the color of the masks as quickly as possible. Longer reaction times are interpreted as increased attention capture and higher sensitivity to the sexual or neutral cues. During the first week of drug treatment, physiological and subjective sexual responding to erotic film were assessed in an at-home psychophysiological experiment. During subsequent weeks, women rated the pleasantness and intensity of each sexual event after self-administration of the medication. Also, they completed a weekly diary on sexual desire, arousal, and improvement in desire.

Based on the emotional Stroop task, women were divided in a group with low sensitivity to sexual cues (reaction times following neutral words longer than after erotic words) and women with high sensitivity to sexual cues (reaction times following neutral words shorter than following erotic words). In the analyses of the drug effect, this distinction in sensitivity to sexual cues was taken into account. It appeared that, only in the group with low sensitivity to sexual cues (*n* = 29), the use of T + sildenafil resulted in significantly stronger genital and subjective sexual arousal and in higher mean sexual satisfaction during sexual events, compared to placebo [30]. In a separate publication, the effects of T + buspiron were reported [29]. In the analyses of these drug effects, women were divided in a group showing low and a group showing high sexual inhibition. The selection of the high inhibition group was based on low sexual satisfaction and arousal scores in the T + sildenafil condition. The high inhibition group (*n* = 28) showed significantly stronger genital and subjective arousal and higher sexual satisfaction and stronger sexual improvement in the T + buspiron condition compared to placebo. The reported side effects were mild, blushing and headache in the T + sildenafil condition and dizziness in the T + buspiron condition.

The initial results with these drugs seem promising. However, the study groups were small, and—although crucial—the validity and reliability of making accurate distinctions between women with high and low sexual sensitivity or between those with high and low sexual inhibition have not been established. At the company’s website, it is stated that an innovative classification system based on genetic markers has been developed and that a phase III study is being prepared. However, to date, no further publications on this classification system or on further drug trials have appeared.

### Bremelanotide

Bremelanotide (BMT) is a melanocortin-receptor agonist with the potential to stimulate dopamine in the medial preoptic area (MPOA), a locus implicated in the sexual behavior of several species. In hormone-primed female rats, infusion of BMT into the MPOA increases sexual appetitive behaviors towards males, such as solicitations, hops and darts, and mounting [31]. In a phase II trial, the safety and efficacy of three different doses (0.75, 1.25, or 1.75 mg) of subcutaneous BMT were tested in premenopausal women with FSIAD [32]. Over a 12-week period, women could give themselves, at home and on demand, an injection with BMT into the anterior thigh or abdomen, approximately 45 min prior to anticipated sexual activity. Outcome measures were the change from baseline in number of SSEs during the last 4 weeks of treatment, sexual desire and arousal domain scores on the Female Sexual Function Index, and scores on distress about lack of desire and arousal.

In the reported analyses, the data of the two groups receiving the highest doses were pooled and compared with placebo. Apparently, the lowest dose of BMT showed no effect at all. The mean change in SSEs was 0.7 in the BMT group, which was significantly higher than 0.2 in the placebo group. Also, sexual function and sexual distress scores showed stronger improvement in the BMT group compared to placebo. The most common reported side effects were nausea, flushing, and headache. Nausea was reported in 22% of BMT users, compared to 3% in placebo users, but very few BMT users ended participation in the study because of this side effect. Also, 24% of the BMT group reported injection-site reactions, such as irritation, rash, or swelling. No serious BMT-related side effects were reported. Physical examinations showed a small increase in blood pressure and a decrease in heart rate 4 h post BMT dose. Taken together, the results indicate an increase relative to placebo of 0.5 satisfying sexual event a month. The authors describe this finding as a clinically significant improvement; however, the effect is even smaller than the reported effect of flibanserin.

### Testosterone Intranasal Gel

A new drug under investigation is TBS-2, a testosterone intranasal gel [33]. Effects of the gel on genital and subjective sexual arousal in response to an erotic film were tested in women with HSDD or anorgasmia. Effects of the gel were compared with the Intrinsa (testosterone) patch in the HSDD group and with placebo in the anorgasmia group. In women with HSDD, significantly stronger feelings of sexual arousal, sensuality, and positive affect were observed in those receiving TBS-2 compared to the Intrinsa patch. In women with anorgasmia, a significantly stronger genital response (measured with vaginal photoplethysmography) compared to placebo was observed 30 min after administration in the TBS-2 high-dose group and 4.5 h after administration in the TBS-2 low-dose group. No safety concerns were reported and plasma testosterone levels did not exceed the upper limit of normal levels. In a subsequent study, the effect of the gel was tested in women with anorgasmia [34]. Effects on genital and subjective sexual response and occurrence of orgasm during clitoral vibrotactile stimulation in combination with an erotic film were tested. Relative to placebo participants, TBS-2-treated participants reported significantly stronger feelings of sexual arousal and showed stronger genital response as measured by vaginal photoplethysmography. Four women in the TBS-2 group and 2 in the placebo group experienced orgasm; this difference was, however, not significant. Whether TBS-2 will undergo further study is not reported.

### BP101

Another new drug under investigation is BP101, a synthetic peptide molecule. The structure has not been disclosed for commercial reasons. The effect of the drug on sexual behavior was tested in three experiments in female rats [35], one experiment on the acute effects of a single intranasal administration, one on the effects of long-term (16 days) daily intranasal administration, and one on the effects of administration directly into the brain, more specifically into the olfactory bulb, the ventral medial hypothalamus, the MPOA, or the ventral tegmental area. Effects on solicitation, lordosis, and social approach behaviors, as well as genital sniffing, were studied. In experiments 1 and 2, an effect of BP101 was observed for solicitation behavior only. In experiment 3, it was observed that administration of BP101 in the MPOA and not in other brain areas increased solicitation behaviors. BP101 is currently undergoing randomized, double-blinded, placebo-controlled phase II studies. Safety, tolerability, pharmacokinetics, pharmacodynamics, and effects on sexual function will be studied after multiple-dose administration of BP101 nasal spray in healthy volunteers (https://clinicaltrials.gov/ct2/show/NCT03102489) and in women with HSDD (https://clinicaltrials.gov/ct2/show/NCT03080298).

### D-cycloserine

A potentially interesting new approach to psychopharmacological treatment of FSIAD may be the use of memory-enhancing medication in combination with cognitive behavioral therapy (CBT). The combination of CBT with d-cycloserine (DCS), a partial agonist of the NMDA receptor which is essential in learning and memory, has already been investigated in the context of anxiety disorders and indicates that DCS can facilitate effectiveness of extinction-based therapy [36]. Besides extinction of fear responses, there is also evidence that DCS can enhance extinction learning of responses to appetitive stimuli, such as drugs [37, 38]. In contrast to the compounds described in the previous sections, which aim is to increase neurobiological sensitivity to sexual cues, the aim of DCS will be to facilitate the basic learning processes underlying CBT interventions in the treatment of sexual dysfunctions. Pharmacological enhancement of the acquisition of positive associations with sex may facilitate the efficacy of CBT for FSIAD, while enhancing memory for extinction of unwanted sexual responses may be useful in CBT for hypersexuality. Recently, we investigated the potential effect of DCS on the extinction of Pavlovian-conditioned sexual responses [39•]. In this placebo-controlled study in healthy female participants, a classical conditioning procedure was followed, involving repeated pairing of an erotic picture (the conditional stimulus, CS) with clitoral vibrostimulation (the unconditional stimulus, US). After this conditioning procedure, the CS was presented repeatedly without the US, to extinguish the conditioned responses. Following this extinction procedure, the women received DCS or placebo. The next day, responses to the CS were tested. Relative to the placebo group, the DCS group showed weaker sexual responses to the CS, indicating enhanced extinction of conditioned sexual responses due to DCS administration. Currently, we are investigating the potentially facilitating effect of DCS on the acquisition of conditioned sexual responses (Van Veen, Brom, Weijenborg, Both, in preparation). In this study, again, an erotic picture is repeatedly paired with clitoral vibrostimulation, and now this acquisition procedure is immediately followed by administration of DCS or placebo. It is expected that the next day, conditioned sexual arousal responses to the CS will be stronger in the DCS group. If DCS shows to facilitate sexual learning, it may offer an interesting method to enhance effectivity of CBT for FSIAD. Administration of DCS following positive sexual experiences that are instigated during CBT—for example following pleasurable individual or couple sensate focus exercises—may facilitate memory for sexual reward and through that, sexual interest and arousal.

### Concluding Remarks

It is likely that in the future, apart from flibanserin, more psychopharmacological treatments for FSIAD will reach the market. Although pharmacological enhancement of sexual responsiveness by facilitation of sexual excitatory or diminishment of sexual inhibitory processes may be helpful in the treatment of FSIAD, to date, the observed effects of flibanserin and other new drugs seem limited in terms of clinical significance (see Table 1). It is questionable if women will be willing to use medication, with side effects such as dizziness, nausea, or headache and unknown long-term use effects, to increase the number of satisfying sexual encounters to an additional once a month or less. It should also be noted that pharmacological facilitation of sexual interest and arousal may be more successful when treatment also focuses on psychological and relational factors of the sexual problem. As emphasized by the incentive motivation model, sexual interest and arousal result from an interaction between a sensitive sexual response system and incentives that activate this system. The sensitivity of the sexual response system plays a role in conjunction with the meaning and intensity of the real or imaginary stimuli. When a woman has predominantly negative sexual experiences, as a result of sexual abuse for example, sexual stimuli may activate negative emotions such as fear or disgust, instead of feelings of interest, arousal, and pleasure. Facilitation of sexual responsiveness will not change the negative meaning of sex as stored in memory. CBT to diminish fear or disgust and sex therapy interventions to acquire positive associations with sex will likely be more helpful [40]. Furthermore, when there are conflicts in the relational context, poor communication, or a lack of intimacy, a woman may be reluctant to respond to sexual stimulation. Also, due to a lack of sexual knowledge and skills, sexual activity may not be very rewarding. Under these circumstances, stimulation of sexual responsiveness with medication cannot be expected to have much positive effect. Therefore, it should be strongly recommended to treat the couple rather than only the woman with low sexual interest and arousal problems and to embed psychopharmacological intervention in cognitive behavioral and sex therapy interventions [41•].

**Table 1:** Recently investigated drugs aimed at increasing female sexual desire and arousal

Drug	Systems targeted	Studies	Participants, *n*	Usage and side effects	Effects	Current status
Flibanserin	Serotonergic and dopaminergic	21, 22, 32, 24	Pre- and postmenopausal women with HSDD, *n* = 5914 in metanalysis [24••]	Daily, oral, 100 mg Dizziness, somnolence, nausea, fatigue.	Across the phase 3 studies, increase relative to placebo of 0.5 SSEs a month and significantly higher sexual desire scores [24••]	Approved by American Food and Drug Administration
Testosterone combined with sildenafil	Central androgen receptors and phosphodiesterase-mediated genital sexual response	28	Pre- and postmenopausal women with HSDD or FSAD and low sensitivity for sexual cues, *n* = 29	On demand, testosterone 0.5 mg sublingual plus sildenafil, 50 mg oral, 4 h prior to anticipated sexual activity Blushing and headache	Significantly stronger genital and subjective sexual arousal, higher mean sexual satisfaction during sexual events compared to placebo	Under investigation
Testosterone combined with buspiron	Central androgen receptor and serotonin_1A_ receptor	29	Pre- and postmenopausal women with HSDD or FSAD and high sexual inhibition, *n* = 28	On demand, testosterone 0.5 mg sublingual plus buspiron, 10 mg oral, 4 h prior to anticipated sexual activity Dizziness	Significantly stronger genital and subjective arousal and higher sexual satisfaction during sexual events compared to placebo	Under investigation
Bremelanotide	Melanocortin-receptor agonist with the potential to stimulate dopamine system	33	Premenopausal women with FSIAD Placebo group *n* = 99, low-dose group *n* = 100 medium-dose group *n* = 99 high-dose group *n* = 99	On demand, 0.75, 1.25, or 1.75 mg, self-administered subcutaneous injection into the anterior thigh or abdomen, 45–60 min before anticipated sexual activity Nausea, flushing, headache	Pooled results of the two highest-dose groups: significantly higher increase in monthly SSEs compared to placebo (*M* = 0.7 vs. *M* = 0.2) and stronger improvement in sexual function and sexual distress scores	Under investigation
TBS-2	Central androgen	34, 35	Study 1: women with HSDD, *n* = 16, and anorgasmia, *n* = 16 Study 2: women with anorgasmia, *n* = 59	Single-dose testosterone, intranasal gel	Study 1: significantly stronger increase in VPA compared to placebo in the anorgasmia group and significantly stronger feelings of sexual arousal and sensuality compared to Intrinsa in the HSDD group Study 2: more intense feelings of sexual arousal after sexual stimulation and stronger increase in VPA compared to placebo	Under investigation
BP101	Synthetic peptite molecule, target not disclosed	36	Female rats Experiment 1: placebo *n* = 9, 4-dose groups *n* varies from 11 to 12 Experiment 2: Daily intranasal, placebo *n* = 16, dose groups *n* varies from 15 to 19 Experiment 3: Injected in brain, *n* varies from 5 to 17	Single intranasal or 16 days daily intranasal, or administration directly into the brain	Experiment 1 and 2: significantly more solicitation behavior compared to placebo Experiment 3: administration in MPOA significantly increased solicitation behavior	Under investigation

*HSSD* hypoactive sexual desire disorder, *SSEs* sexual satisfying events, *FSAD* female sexual arousal disorder, *MPOA* medial preoptic area
